# Global incidence in hospital-associated infections resistant to antibiotics: An analysis of point prevalence surveys from 99 countries

**DOI:** 10.1371/journal.pmed.1004178

**Published:** 2023-06-13

**Authors:** Ruchita Balasubramanian, Thomas P. Van Boeckel, Yehuda Carmeli, Sara Cosgrove, Ramanan Laxminarayan

**Affiliations:** 1 One Health Trust, New Delhi, India; 2 Health Geography and Policy Group, ETH Zurich, Zurich, Switzerland; 3 Division of Epidemiology, Tel Aviv Sourasky Medical Center, Tel Aviv, Israel; 4 The Johns Hopkins University School of Medicine, Baltimore, Maryland, United States of America; 5 Princeton University, Princeton, New Jersey, United States of America

## Abstract

**Background:**

Hospital-associated infections (HAIs) are an important cause of morbidity and mortality around the world. Many HAIs are caused by drug-resistant bacterial pathogens, but there are major gaps in our understanding of the number of hospital-associated drug-resistant infections (HARIs) worldwide. As such, we estimated trends in prevalence of HARIs caused by high priority pathogens (*Escherichia coli*, *Acinetobacter* spp., *Klebsiella* spp., *Staphylococcus aureus*, *Enterobacter* spp., and *Pseudomonas* spp.) in 195 countries.

**Methods and findings:**

Resistance prevalence estimates were extracted from 474-point prevalence surveys (PPS) from 99 countries published between 2010 and 2020 coupled with country-level estimates of hospitalization rates and length of stay. Prevalence estimates were transformed in yearly incidence of HARIs per year by country and income group. We estimate the global number of HARIs per year to be 136 million (95% credible interval (CI) 26 to 246 million) per year, with the highest burden in China (52 million, 95% CI 10 to 95 million), Pakistan (10 million, 95% CI 2 to 18 million), and India (9 million, 95% CI 3 to 15 million). Among income groups, middle-income countries bore the highest burden of HARIs per year (119 million, 95% CI 23 to 215 million). Our analysis was constrained by the limited number of PPS for HARIs, lack of community-associated data on antibiotic-resistant infections, and our population level analysis.

**Conclusions:**

In this study, we observe, in the absence of systematic surveillance systems for HARIs, a baseline overview of their rates. Our yearly estimates highlight the global threat of HARIs and may help define strategies to tackle resistance in hospital settings.

## Abstract 2

Ruchita Balasubramanian and colleagues, estimate the incidence of hospital-associated infections resistant to antibiotics in 195 countries using point prevalence survey data from across the globe.

## Author summary

**Why was this study done?:**

- Low- and middle-income settings (LMICs) that have poor antimicrobial stewardship and limited microbiology diagnostic capacities are grossly affected by hospital-associated drug-resistant infections (HARIs).
- In the absence of robust national surveillance networks in LMICs to appropriately identify the threat of HARIs, we have identified a critical need to quantify the burden of HARIs in low-, middle-, and high-income countries, specifically those caused by common pathogens including *E*. *coli*, *Klebsiella* species (spp.), *Acinetobacter* spp., *Pseudomonas* spp., *Staphylococcus* spp, and *Enterobacter* spp.

**What did the researchers do and find?:**

- We extracted data from point prevalence surveys (PPS), conducted over 10 years across 195 countries, coupled with country-level estimates of hospitalization rates and durations.
- We analyzed the data to illustrate global trends in HARIs based on country, income group, and pathogen that could help to focus public health intervention.
- We estimate the global number of HARIs per year to be 136 million, with the highest burden in China, Pakistan, and India, emphasizing the need for ongoing surveillance.
- Compared to other income groups, middle-income groups demonstrate the highest burden of HARIs per year.

**What do these findings mean?:**

- Although our study was limited by the number of PPS for HARIs, lack of community-associated data on antibiotic-resistant infections, and our population analysis, in the absence of systematic surveillance systems for HARIs, we provide a baseline overview of their rates.
- While antimicrobial resistance (AMR) is not currently recognized as a challenge on par with the “big three” including HIV, TB, and malaria, our results show that the burden of HARIs is substantial and is thus worthy of further recognition by world leaders.

## Introduction

Hospital-associated infections (HAIs) are a common cause of morbidity and mortality [1], in particular among vulnerable patients [2]. Many HAIs caused by common bacterial pathogens are no longer treatable with affordable first-line antibiotics, such as penicillins and cephalosporins [3,4]. For example, β-lactam resistance in bacteria such as *Escherichia coli* has been documented over the past 2 decades [5,6]. Resource poor settings, especially in low- and middle-income countries (LMICs), have poor antimicrobial stewardship and limited microbiology diagnostic capacities [1,7]. As such, they are disproportionately affected by hospital-associated drug-resistant infections (HARIs) or hospital-diagnosed infections within individuals in a hospital setting that were resistant infections. Nevertheless, in many LMICs, national surveillance systems for HARIs are nascent or entirely absent.

Long-term investments in improving surveillance of antimicrobial resistance (AMR) will involve strengthening laboratory capacity and training human resources including laboratory personnel, a process that could take years, if not decades. Notably, the Global Antimicrobial Resistance and use Surveillance System (GLASS) has attempted to monitor AMR in common bacteria as well as documenting human antibiotic consumption. While 109 countries and 2 territories were enrolled in GLASS-AMR and 99 countries and territories provided information on the status of AMR surveillance implementation, there was no evidence for an increase in global testing coverage [8]. In the short term, estimating the number of HARIs per year in each country can help identify funding priorities, as well as inform antibiotic research and development priorities.

Point prevalence surveys (PPS) have been used to infer incidence rates of HAIs in Europe and the United States [9–12] and could help set priorities for surveillance of HARIs in LMICs, including making international comparisons. However, the inference of global trends in HARIs from PPS is challenging for at least 3 reasons. Firstly, PPS on HAIs and HARIs may not have been conducted in all locations around the world. Thus, we are required to extrapolate infection rates by income groups. Second, even if HARIs are reported in a particular location, the number of hospital visits and length of stay must be concurrently reported to generate estimates of HARI prevalence, which may be difficult to estimate. Thirdly, most estimates are available at the hospital level rather than at the country level. Carefully extrapolating these estimates to the country level is dependent on the availability of hospitalization rate data.

A recent study attempted to estimate the global burden of bacterial AMR in 2019, reporting an estimate of 4.95 million deaths associated with bacterial AMR and 1.27 million deaths attributable to bacterial AMR [13]. While this represents the first comprehensive overview of the global burden of AMR using statistically complex methodology, it does not distinguish between hospital and community-associated settings and uses an intractable approach that introduces some difficulty in the verification of the burden estimated [13]. Moreover, the investigators failed to estimate the number of hospital-acquired and community-acquired infections in which the estimated deaths occurred. In this study, we used resistance prevalence extracted from 474 hospital-based PPS and published between 2010 and 2020 along with country-level estimates of hospitalization rates and durations. We combined this with statistical extrapolation based on common economic indicators to estimate the number of HARIs per year by country and by country income group. Utilizing our simple approach, we are able to provide global and national estimates of the number of HARIs.

## Methods

We screened the bibliographic databases Pubmed and Google Scholar for PPS published between January 2010 and December 2020 that reported prevalence of drug-sensitive and drug-resistant infections for common bacterial pathogens: *E*. *coli*, *Klebsiella* species (spp.), *Acinetobacter* spp, *Pseudomonas* spp, *Staphylococcus* spp, and *Enterobacter* spp. These bacterial groups were selected based on the availability of data on these pathogens, the documented resistance acquisition and clinical relevance in establishing nosocomial infections, the potential existence of preclinical vaccines, and their classification as critical or high priority on the WHO priority pathogen list [6,14–16]. For all 195 countries and for each of the aforementioned pathogens, the following search terms were used: ((((((((antimicrobial) OR (antibiotic)) AND (resistance)) AND (hospital associated)) AND (infection)) AND (human)) AND (pathogen)) AND (country)) AND (("2010/01/01"[Date—MeSH]: "2020/12/01"[Date–MeSH)), resulting in a total of 1,170 distinct abstracts (see Fig A in S1 Text for overview of systematic literature review). Studies were excluded if they had not been published within the 10-year timeframe, if antibiotic resistance proportions for isolates taken from patients exhibiting HAIs were not reported, or if isolates were taken from patients in community settings or combined with those from patients exhibiting HAIs.

Antibiotic resistance prevalences were reported for 122 antimicrobials. These were categorized into the following 13 classes: tetracyclines, penicillins, sulfonamides, macrolides, monobactams, aminoglycosides, quinolones, cephalosporins, amphenicols, carbapenems, polymixins, glycopeptides, and “others” (Table A in S1 Text).

For each survey, we extracted the digital object identifier (DOI) of the publication, the first author’s last name, the publication date in years, the country ISO3 code, the latitude-longitude decimal coordinates of the geographical center of mass of the survey, the start and end dates of the survey, and the number of beds of the hospital(s) where the survey was conducted. Pathogen-specific information including the pathogen causing the infections, the strain of the pathogen, the type of infection (bronchitis, urinary tract infection, etc.), the sample type (blood, urine, fecal, etc.), the number of infections reported, the prevalence of resistance for each antibiotic–pathogen combination tested, the antibiotic compounds tested, and the associated Anatomical Therapeutic Chemical Classification System (ATCCS) code were also included. See supporting information for a more complete description.

We used country-level indicators to estimate hospitalization rates per capita or the number of hospitalizations that occur per population per year. For 78 countries, hospitalization rates were obtained preferentially from OECD and if this was not available Eurostat hospital discharge rates and country-specific sources, such as ministries of health were collected (Table B in S1 Text). Hospitalization rates were collected between 2010 and 2019 if possible and averaged over the years for which data was available. For an additional 26 countries where we were unable to identify hospitalization rates from published databases or resources, hospitalization rates were inferred from the 78 countries that we did have hospitalization rates for using a logistic regression model. This model contained the following explanatory variables after excluding collinear variables: gross domestic product (GDP) per capita (in current US $) (variance inflation factor (VIF) = 1.75), crude death rate per 1,000 people (VIF = 2.60), number of physicians per 1,000 people (VIF = 3.03), hospital beds per 1,000 people (2.34), percentage of population using safe drinking water sources (VIF = 2.34), percentage of urban population (VIF = 1.05), poverty headcount ratio at US$1.90 per day (VIF = 1.92), and life expectancy at birth (VIF = 1.92). All explanatory variables were obtained from the World Bank [17]. These variables were selected using subject matter knowledge and their reported influence on country-level hospitalization rates [18]. Using World Bank Indicators guarantees that if there was any error in calculating these covariates, this error would be systematic since they use the same methodology from the World Bank. We additionally used VIFs lower than 10 as criteria for the inclusion of explanatory variables. For the remaining countries, we used the median hospitalization rates for the country’s income group: low-income (LIC), low-middle-income (LMIC), high-middle-income (HMIC), or high-income (HIC), where income status is defined in accordance with the 2019 definition from the World Bank [19].

For each country, we estimated the number of HARIs per year (*N*_*DR*,*Y*_) as the product of 3 terms: (1) the population size, *Pop*; (2) the proportion of the population hospitalized each year *Hr*; and (3) the probability that a patient was hospitalized for an antimicrobial-resistant infection.

$$N_{DR,Y}=\overset{1}{\overset{︷}{Pop}}\times \overset{2}{\overset{︷}{H_{r}}}\times \overset{3}{\overset{︷}{\sum_{j=1}^{H}\frac{\sum_{i=1}^{D}\;\sum_{k=1}^{d}\frac{N_{i,j}\times P_{r,i,k,j}}{V_{j}}}{H}\times \frac{1}{T}}}$$

$$V_{j}=\left(\frac{B_{j}\times O}{LOS}\right)\times T,$$

where *Pop* is the population of the country, *H*_*R*_ is the proportion of the population hospitalized each year, and *H is* the number of hospitals for which data on antibiotic-resistant infections could be identified per country. *D* is the number of pathogens considered in the assessment, and *d* is the number of antibiotics to which resistance is reported for each pathogen. *N*_*i*,*j*_ is the number of infections caused by pathogens *i* in hospital *j*. *V*_*j*_ is the number of visits in hospital *j*. *P*_*r*,*i*,*k*_ is the percentage of resistance for the pathogen–drug combination *i-k*. *Bj* is the number of beds in hospital *j*, *T* is the period of data collection in days, LOS of the average length of stay in hospital, and *O* is the occupancy rate of hospitals per country according to income group.

We quantify the uncertainty (credible intervals (CI)) associated with estimates of the global number of antibiotic-resistant infections per year using Monte Carlo simulations (1,000 draws). We used Latin hypercube sampling [20] with uniform priors (U) on the length of stay (LOS) and the level of bed occupancy (%) by income group (O), as well as the exclusion of extremely high values of antibiotic-resistant infection rates per hospital visit, based on percentile. The parameter distributions were defined as follows: LOS~U(2 days,10 days); O~U(60%,100%) in high-income countries, O~U(80%,100%) in middle-income countries, O~U(100%,150%) in low-income countries. Percentiles of infection rates per hospitals excluded ~U(98th,100th).

For 43 countries where no more than 2 reports of antibiotic-resistant infections could be identified, or where 2 resistance rates could not be obtained for specific antibiotic-pathogens, we used multivariate imputation by chained equations (MICE) using the default predictive-mean matching method [21] to infer the missing rates using mean rates of antibiotic-resistant infection per hospital to a country’s corresponding income group, which in turn acts as a cross-validation built into the procedure to ensure that extrapolations are as valid as possible.

## Results

We identified 474 publications that reported antibiotic resistance proportions for hospital-associated infections in 99 countries. We extracted 15,723 resistance proportions pooled in six groups of pathogens: *E*. *coli* (*n* = 4,014), *Klebsiella* spp. (*n* = 3,524), *Staphylococcus* spp. (*n* = 2,979), *Acinetobacter* spp. (*n* = 2,296), *Pseudomonas* spp. (*n* = 1,791), and *Enterobacter* spp. (*n* = 1,119).

**Fig 1:**
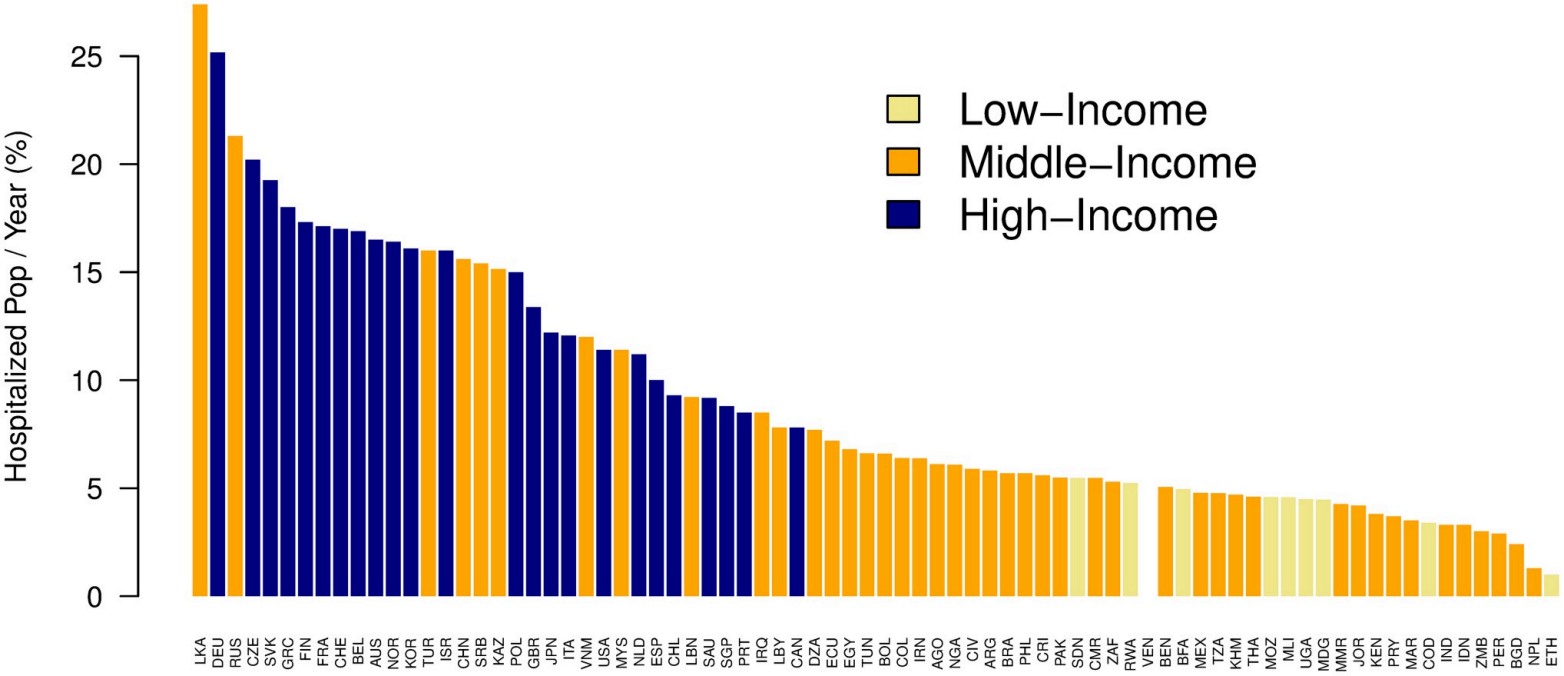
Hospitalizations per year, as percentage of population, in countries with more than 5 million inhabitants. The Y-axis represents the hospitalized proportion of a country’s population per year, represented as a percentage. The X-axis shows the country 3-letter abbreviations. Yellow bars represent low-income countries, orange bars represent middle-income countries, and blue bars represent high-income countries. LKA = Sri Lanka, DEU = Germany, RUS = Russia, CZE = Czech Republic, SVK = Slovakia, GRC = Greece, FIN = Finland, FRA = France, CHE = Switzerland, BEL = Belgium, AUS = Australia, NOR = Norway, KOR = South Korea, TUR = Turkey, ISR = Israel, CHN = China, SRB = Serbia, KAZ = Kazakhstan, POL = Poland, GBR = Great Britain, JPN = Japan, ITA = Italy, VNM = Vietnam, USA = United States of America, MYS = Malaysia, NLD = Netherlands, ESP = Spain, CHL = Chile, LBN = Lebanon, SAU = Saudi Arabia, SGP = Singapore, PRT = Portugal, IRQ = Iraq, LBY = Libya, CAN = Canada, DZA = Algeria, ECU = Ecuador, EGY = Egypt, TUN = Tunisia, BOL = Bolivia, COL = Colombia, IRN = Iran, AGO = Angola, NGA = Nigeria, CIV = Cote d’Ivoire, ARG = Argentina, BRA = Brazil, PHL = Philippines, CRI = Costa Rica, PAK = Pakistan, SDN = Sudan, CMR = Cameroon, ZAF = South Africa, RWA = Rwanda, VEN = Venezuela, BEN = Benin, MEX = Mexico, TZA = Tanzania, KHM = Cambodia, THA = Thailand, MOZ = Mozambique, MLI = Mali, UGA = Uganda, MDG = Madagascar, MMR = Myanmar, JOR = Jordan, KEN = Kenya, PRY = Paraguay, MAR = Morocco, COD = Democratic Republic of the Congo, IND = India, IDN = Indonesia, ZMB = Zambia, PER = Peru, BGD = Bangladesh, NPL = Nepal, ETH = Ethiopia.

Based on our estimates, the countries with the highest hospitalization rates were Sri Lanka, Germany, Russia, Czech Republic, and Slovakia (Fig 1). The average hospitalization rates were 3% across all low-income countries, 6% across all middle-income countries, and 11% across all high-income countries.

**Fig 2:**
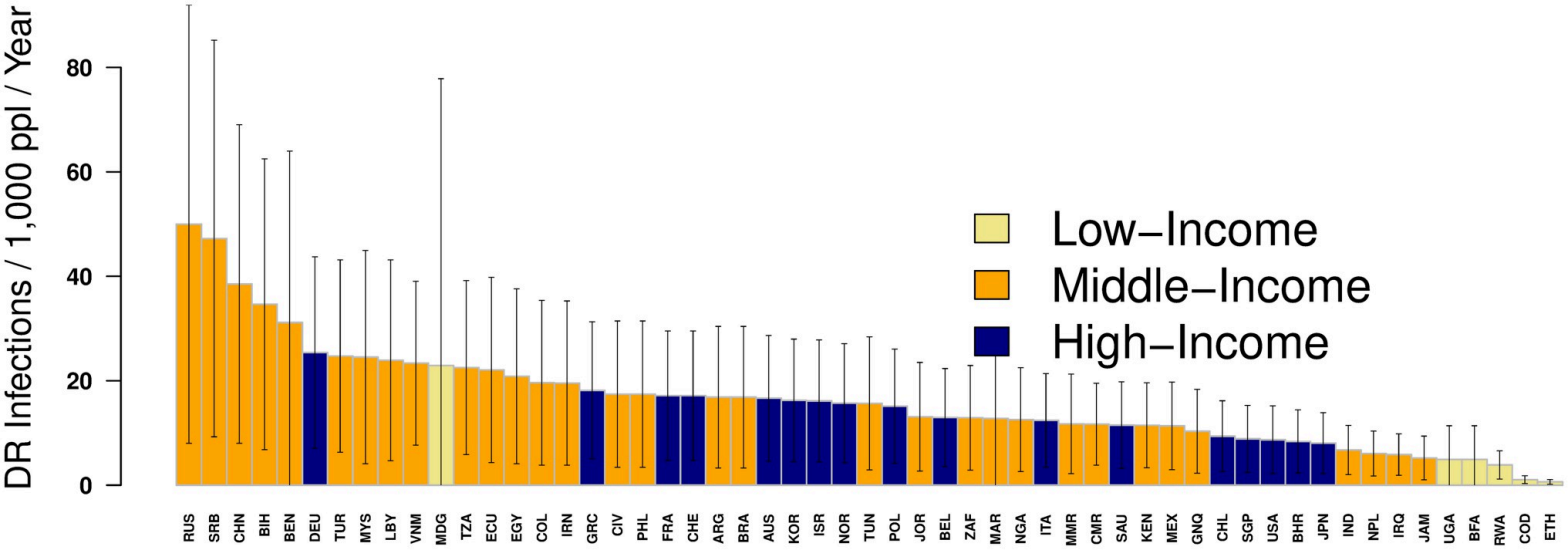
Hospital-associated antibiotic-resistant infections per 1,000 population per year in countries with at least 2 surveys reporting drug resistance proportions. The Y-axis represents the number of antibiotic-resistant infections per 1,000 country population per year from 2010–2020. Bars represent 95% CIs. The X-axis shows the country 3-letter abbreviations. Yellow bars represent low-income countries, orange bars represent middle-income countries, and blue bars represent high-income countries. Vertical bars represent 95% CIs. RUS = Russia, SRB = Serbia, CHN = China, BIH = Bosnia and Herzegovina, BEN = Benin, DEU = Germany, TUR = Turkey, MYS = Malaysia, LBY = Libya, VNM = Vietnam, MDG = Madagascar, TZA = Tanzania, ECU = Ecuador, EGY = Egypt, COL = Colombia, IRN = Iran, GRC = Greece, CIV = Cote d’Ivoire, PHL = Philippines, FRA = France, CHE = Switzerland, ARG = Argentina, BRA = Brazil, AUS = Australia, KOR = South Korea, ISR = Israel, NOR = Norway, TUN = Tunisia, POL = Poland, JOR = Jordan, BEL = Belgium, ZAF = South Africa, MAR = Morocco, NGA = Nigeria, ITA = Italy, MMR = Myanmar, CMR = Cameroon, SAU = Saudi Arabia, KEN = Kenya, MEX = Mexico, GNQ = Equatorial Guinea, CHL = Chile, SGP = Singapore, USA = United States of America, BHR = Bahrain, JPN = Japan, IND = India, NPL = Nepal, IRQ = Iraq, JAM = Jamaica, UGA = Uganda, RWA, Rwanda, COD = Democratic Republic of Congo, ETH = Ethiopia.

In countries where at least 2 surveys on hospital-associated drug resistance were conducted (*n* = 56), those with the highest HARI rates (HARIs per year) were Russia, Serbia, China, Madagascar, Bosnia, and Benin (Fig 2). The high-income countries with the most HARIs were Germany and Greece, ranking 6th and 17th globally, respectively.

Using imputation of resistance rates per hospital visit by income group, we estimate the global number of HARIs per year at 136 million (95% CI 26 to 246 million) per year.

**Fig 3:**
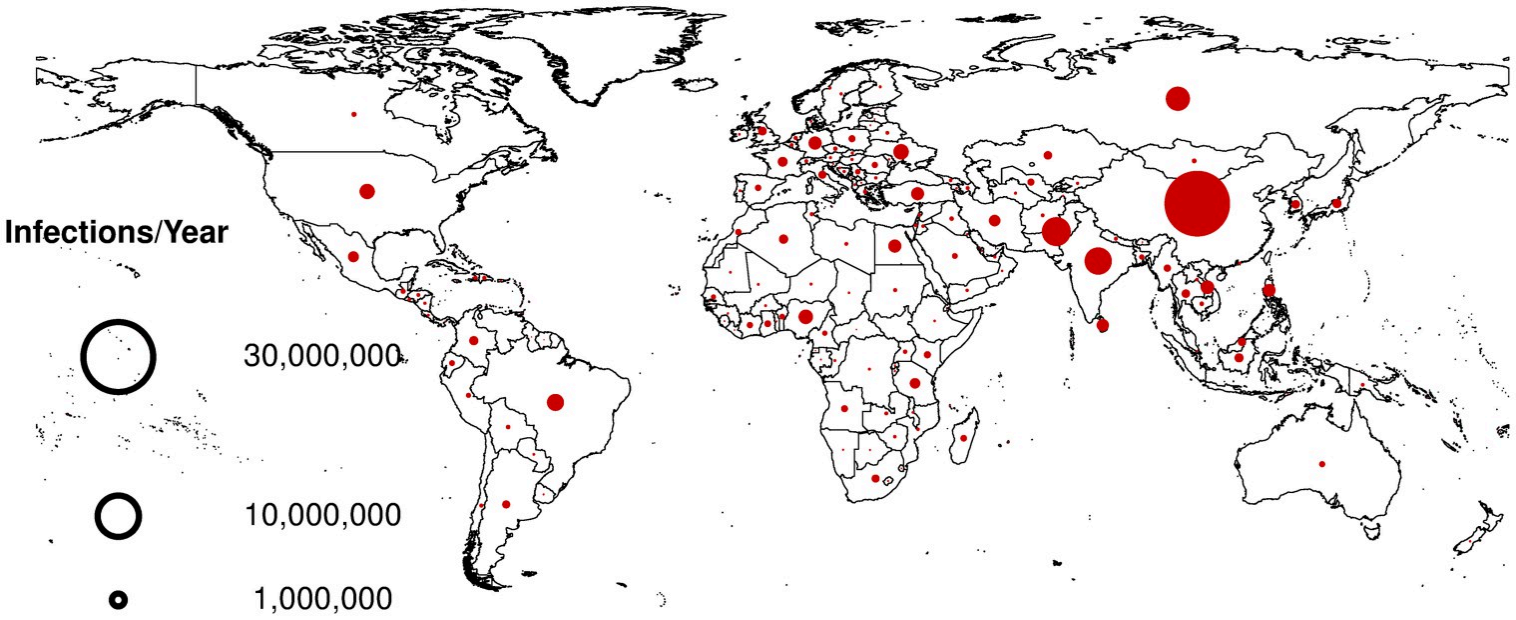
Global distribution of hospital-associated antibiotic-resistant infections per year. Size of circles are proportional to the number of hospital-associated antibiotic-resistant infections per year. The figure legend includes 3 circle sizes denoting 3 reference points for the number of hospital-associated antibiotic-resistant infections per year. The direct link to the base layer of the map can be found at: https://gadm.org/. The license for the GDAM map used can be found at: https://gadm.org/license.html.

In absolute terms, the countries with the highest number of HARIs per year were China (52 million, 95% CI 10 to 95 million), which far outnumbered the burden in its region, Pakistan (10 million, 95% CI 2 to 18 million), India (9 million, 95% CI 3 to 15 million), Russia, (7 million, 95% CI 1 to 13 million), Brazil (4 million, 95% CI 1 to 6 million), Ukraine (3 million, 95% CI 0 to 5 million), United States (3 million, 95% CI 1 to 5 million), and Nigeria (2 million, 95% CI 1 to 5 million) (Table C in S1 Text). The high-income country with the most HARIs per year was the United States (Fig 3 and Table C in S1 Text).

Middle-income countries (Table 1) had more HARIs per year (119 million, 95% CI 23 to 215 million) than low-income (2 million, 95% CI 0 to 5 million) and high-income countries (15 million, 95% CI 4 to 25 million). In low-income countries, the HARI burden was dominated by *E*. *coli*, *Acinetobacter* spp., and *Staphylococcus* spp.; the burden of *Klebsiella* spp. and *Enterobacter* spp. was low. In middle-income countries, the burden was dominated by *Acinetobacter* spp. and *E*. *coli*, with an increase in *Klebsiella* spp. Similarly, among high-income countries, *Acinetobacter* spp., *E*. *coli*, and *Klebsiella* spp. dominated the HARI burden, while *Staphylococcus* spp. infections represented a smaller share (Table 1).

**Table 1:** Hospital-associated antibiotic-resistant infections per year by income group and bacterial pathogen type.

**Total high-income countries**	**14,775,000 (4,000,000–25,000,000)**
*E*. *coli*	3,398,250 (1,000,000–6,000,000)
*Acinetobacter* spp.	3,102,750 (1,000,000–6,000,000)
*Staphylococcus* spp.	2,068,500 (500,000–4,000,000)
*Klebsiella* spp.	2,659,500 (1,000,000–5,000,000)
*Enterobacter* spp.	2,511,750 (1,000,000–5,000,000)
**Total middle-income countries**	**119,202,000 (23,000,000–215,000,000)**
*E*. *coli*	25,032,420 (5,000,000–45,000,000)
*Acinetobacter* spp.	23,840,400 (5,000,000–43,000,000)
*Staphylococcus* spp.	21,456,360 (4,000,000–39,000,000)
*Klebsiella* spp.	20,264,340 (4,000,000–39,000,000)
*Enterobacter* spp.	17,880,300 (3,000,000–32,000,000)
**Total low-income countries**	**2,107,000 (0–5,000,000)**
*E*. *coli*	632,100 (0–2,000,000)
*Acinetobacter* spp.	421,400 (0–1,000,000)
*Staphylococcus* spp.	611,030 (0–2,000,000)
*Klebsiella* spp.	105,350 (0–250,000)
*Enterobacter* spp.	147,490 (0–350,000)

The total number of HARIs per year were included for each income group and additionally subdivided for the following bacterial pathogens: *E*. *coli*, *Acinetobacter* spp., *Staphylococcus* spp., *Klebsiella* spp., and *Enterobacter* spp.

## Discussion

International systematic surveillance systems for HARIs have only recently been established and include very few LMICs [22]. However, a baseline estimate of the number of HARIs on a global scale is needed for determining optimal resource allocation among countries and for setting priorities to tackle the rise of AMR in hospitals. Although imperfect as a surrogate for systematic surveillance, PPS can help generate an order-of-magnitude, outer envelope for the total number of HARIs per year. As such, our study utilizes a relatively lean approach to estimate the global number of HARIs per year to guide effective intervention and invest in surveillance for HARIs. The global burden is estimated to be 136 million (95% CI 26 to 246 million) per year, with the highest burden in China (52 million, 95% CI 10 to 95 million), Pakistan (10 million, 95% CI (2 to 18 million), and India (9 million, 95% CI 3 to 15 million). Middle-income countries bore the highest burden of HARIs per year (119 million, 95% CI 23 to 215 million) compared to other income groups.

Comparable approaches have been taken by the Global Point Prevalence Survey of Antimicrobial Consumption and the European Center for Disease Control for AMR in hospitals as well as for estimating AMR prevalence in animals raised for food and to infer trends for other diseases of global importance, including *P*. *falciparum* malaria [22–25].

In this study, we summarized current evidence from PPS on HARIs per year between 2010 and 2020 and estimate an annual incidence of 136 million HARIs per year. This provides a more specific and concerning overview of the resistance situation within high-risk, healthcare-associated settings. As compared to the recent study by Murray and colleagues that had reported a global burden of 4.95 million deaths attributable to bacterial AMR in 2019 alone, aggregated across hospital and community settings, these data might suggest that the problem is greater than originally anticipated [13]. Community-acquired drug-resistant infections are known to pose a nontrivial burden in places like Europe, increasing trends in resistance rates to penicillin and other beta-lactam antibiotics have been observed [26]. While the relative burden of community-acquired drug-resistant infections compared to hospital-acquired drug-resistant infections is not well quantified globally, the fact that community-acquired drug-resistant infections including pneumonia can lead to hospitalization highlights the threat these infections pose beyond the community and within hospital settings [27]. Our results complement these results from Murray and colleagues by describing a substantial burden of hospital-associated resistant infections alone that contribute to the death burden reported. As such, we have highlighted a setting in which drug-resistant disease should be investigated further.

Current evidence suggests that middle-income countries have, by far, the largest burden of HARIs, which is in line with previous studies reporting the burden of deaths associated with bacterial AMR to be highest in South and Southeast Asia. This trend is likely for multiple reasons. A lack of antibiotic stewardship and lack of personal accountability from medical personnel may lead to irrational prescribing behavior, including the unnecessary use of broad-spectrum antibiotics [28]. Although Browne and colleagues cites higher antibiotic consumption in high-income countries compared to South and Southeast Asian countries, South and Southeast Asia also demonstrate a lack of antibiotic stewardship for animal health and wastewater management that act as additional drivers for drug resistance within these regions [28,29]. Collignon and colleagues additionally suggests that antibiotic usage was not the most associated with drug resistance [30]. The high consumption of antibiotics in high-income countries could additionally account for the fact that the second highest burden of HARIs per year were indeed concentrated in high-income settings (15 million, 95% CI 4 to 25 million) [29].

Additionally, in middle-income countries, access to antibiotics is increasing and relatively unrestricted [31], over-the-counter sales are the norm rather than the exception, and people can often afford to buy antimicrobials unlike in many in low-income countries. These factors favor an indiscriminate use of antibiotics and facilitate the spread of resistance. Third, despite considerable variability in laboratory capacity, middle-income countries have sufficient healthcare capacities to allow high hospitalization rates [32,33] and detect HARIs per year. In contrast, in low-income countries, limited income may reduce access to antibiotics, thereby slowing the rise of resistance, and limited diagnostic capacities may mean that drug-resistant infections go undetected. However, the high burden in middle-income countries may reflect a broader problem related to hospital hygiene and high infection rates, resulting in the higher hospitalization rates observed. Recent works suggested that poor hospital hygiene protocol in LMICs (i.e., hand hygiene) led to patients in from LMICs being exposed to rates of healthcare-associated infections at least 2-fold higher than in HIC, [34] and up to 38% of health facilities in LMICs did not have access to soap for hand washing [35]. However, that hypothesis may be challenging to test because poor hygiene conditions are unlikely to be documented systematically.

As with any modeling analysis, our study comes with limitations. These estimates have considerable uncertainty because reports on hospitalization rates and resistance for individual drug–pathogen combinations are scarce. The uncertainty levels are heightened by potential reporting bias that may reflect countries’ divergent laboratory capacities for antimicrobial susceptibility testing. With regards to our analysis, firstly, although we obtained data from countries accounting for >90% of the world population, only 99 countries had reported studies on HARIs, and these data reported are from PPS that may not be nationally representative or instead biased towards larger tertiary care hospitals. Although this is the best information available, the biggest data gap was in low-income countries: just 11 countries in this group reported information, often based on small samples. This, in combination with lack of standardized reporting for hospitalization rates and limited access to molecular diagnostics makes estimating AMR trends for these countries a challenge that calls for better reporting and surveillance efforts [1,7]. Considering that access to antibiotics remains largely unregulated in low-income countries, our estimates may underestimate the burden of HARIs. Secondly, our estimates are based on reports from hospitals and do not include community-associated antibiotic-resistant infections as was done in Murray and colleagues [13]. These infections are potentially even greater in number and health consequence but also more challenging to estimate. Relatedly, we quantify the number of infections associated with hospitalization, but not those caused or acquired during hospitalizations. Indeed, infections could have been contracted from the community, which then could have led to hospitalization if the infection became acute. Previous studies have suggested that gram-negative bacteria associated with hospital-acquired infections have demonstrated higher resistance proportions in low and lower-middle-income countries compared to high-income countries [36]. Although, our study showed the lowest burden of HARIs per year in low-income countries (2 million, 95% CI 0 to 5 million), this could be a consequence of our investigation into hospital-associated rather than hospital-acquired drug-resistant infections, in conjunction with the limited availability of data from low-income countries in our study. Further studies could explore more directly the burden of hospital-acquired resistant infections. Thirdly, our analysis uses PPS reporting resistance at the population level rather than in individual isolates from patients. Therefore, it does not account for the fact that resistance to multiple drug–pathogen combinations may be linked to the same infection event. For this reason, our results may overestimate the global number of drug-resistant infections in healthcare settings. Finally, our analysis was constrained to a limited range of pathogens, but a future direction of this study could involve the investigation of a broader range of pathogens causing hospital-associated resistant infections, in addition to extending the scope of this study beyond the year 2020. Doing so could potentially help capture increasing trends of resistance indicated by available surveillance data that could not be accomplished here.

Without the presence of robust national surveillance networks, we have identified a critical need to quantify the burden of HARIs in low-, middle-, and high-income countries, and more specifically, those caused by high-priority pathogens including *E*. *coli*, *Klebsiella* spp., *Acinetobacter* spp., *Pseudomonas* spp., *Staphylococcus* spp, and *Enterobacter* spp. This study provides an overview of the substantial burden of HARIs and highlights countries and income groups where it demonstrates a particularly high risk. Compared with other diseases of global importance, our estimates suggest that globally, the annual burden of HARIs is not dissimilar to the burden of malaria in terms of the number of infections (228 million) [37]. However, AMR is not currently recognized as a challenge of comparable importance to the “big three” (malaria, TB, and HIV) by international funders and is thus worthy of further recognition by world leaders and public health stakeholders [38].

## Supporting information

S1 Text Supporting information. Fig A. Schematic of literature review and data extraction. Table A. Antibiotic designation and drug class. Table B. Hospitalization rates per country and sources. Table C. Number of HARIs per year by country. Supplementary References.
(DOCX)

## Abbreviations

AMR: antimicrobial resistance
ATCCS: Anatomical Therapeutic Chemical Classification System
CrI: credible interval
DOI: digital object identifier
GDP: gross domestic product
GLASS: Global Antimicrobial Resistance and use Surveillance System
HAI: hospital-associated infection
HARI: hospital-associated drug-resistant infection
LMIC: low- and middle-income country
LOS: length of stay
MICE: multivariate imputation by chained equation
PPS: Point prevalence survey
VIF: variance inflation factor
